# Supporting Risk-Aware Use of Online Translation Tools in Delivering Mental Healthcare Services among Spanish-Speaking Populations

**DOI:** 10.1155/2021/1011197

**Published:** 2021-10-28

**Authors:** Wenxiu Xie, Meng Ji, Mengdan Zhao, Xiaobo Qian, Chi-Yin Chow, Kam-Yiu Lam, Tianyong Hao

**Affiliations:** ^1^Department of Computer Science, City University of Hong Kong, Kowloon, Hong Kong; ^2^School of Languages and Cultures, The University of Sydney, Darlington, Australia; ^3^School of Computer Science, South China Normal University, Guangzhou, China

## Abstract

Neural machine translation technologies are having increasing applications in clinical and healthcare settings. In multicultural countries, automatic translation tools provide critical support to medical and health professionals in their interaction and exchange of health messages with migrant patients with limited or non-English proficiency. While research has mainly explored the usability and limitations of state-of-the-art machine translation tools in the detection and diagnosis of physical diseases and conditions, there is a persistent lack of evidence-based studies on the applicability of machine translation tools in the delivery of mental healthcare services for vulnerable populations. Our study developed Bayesian machine learning algorithms using relevance vector machine to support frontline health workers and medical professionals to make better informed decisions between risks and convenience of using online translation tools when delivering mental healthcare services to Spanish-speaking minority populations living in English-speaking countries. Major strengths of the machine learning classifier that we developed include scalability, interpretability, and adaptability of the classifier for diverse mental healthcare settings. In this paper, we report on the process of the Bayesian machine learning classifier development through automatic feature optimisation and the interpretation of the classifier-enabled assessment of the suitability of original English mental health information for automatic online translation. We elaborate on the interpretation of the assessment results in clinical settings using statistical tools such as positive likelihood ratios and negative likelihood ratios.

## 1. Introduction

Despite the increasing public awareness of the prevalence of mental health issues among populations from low and middle-income countries, accurate, scientific, non-discriminative communication of mental disorders remains a real challenge [1–3]. Within different societal, cultural systems, conventionalised linguistic constructs have been developed over years and decades to describe and convey the underlying social attitudes and understanding of different mental disorders like varieties of anxiety or depressive disorders. In English-speaking multicultural countries, the communication and interpretation of mental disorders and their treatment for non-English-speaking migrant populations pose important challenges to frontline health workers and clinicians [4–6]. The rapid development of machine translation technologies has offered necessary technical means to interact and engage with multicultural vulnerable communities and people who have limited access to mental healthcare services, despite the prevalence of mental health issues among such populations who are at higher risks of developing clinical mental disorders or other comorbidities such as chronic non-communicable diseases or physical health conditions that are likely to exacerbate their mental health issues. Currently, there is very limited research which examines the reliability, safety, or levels of risks in using state-of-the-art online translation tools such as Google Translate in clinical settings for communicating and talking with patients about mental disorders.

Much of current research shows that the use of automatic translation tools in primary healthcare settings is driven by a persistent lack of qualified bilingual health professionals [7–10]. The risk of an unchecked use of translation technologies in specialised health and medical settings which have been developed largely for general cross-lingual communication purposes is real and well documented [11–14]. However, the practical needs for cost-effective translation tools in disease diagnosis and medical treatments are increasing. Although the provision of proper training to a certain number of bilingual health professionals can help reduce healthcare inequality issues, real-life scenarios can be much more complex, uncertain, and dynamic for any health systems at various levels. For example, a recurring issue in clinical settings is the lack of adequately qualified health translators working with under-resourced languages. Even for resourced language pairs such as English-Spanish and English-Chinese, it can be challenging to find bilingual health translators with extensive in-depth knowledge of different medical specialities. That is, the quality of human translation can also be compromised by the complexity and speciality of medical communications. In fact, with simple, direct sentences, online translations tools can fulfil their function to support a meaningful exchange of information between doctors and patients. We argue that health communication technologies like neural translation tools are evolving rapidly and that they have important potential for scaled uptake in health systems, especially those serving vulnerable or disadvantaged communities in under-resourced local health districts. Health technologies can be leveraged to help close the gaps in current medical and healthcare structures and improve the quality and accessibility of healthcare resources to populations and people at risk. Research is needed to develop instruments and aids to improve the safety and reliability of available translation technologies to be adopted in health and clinical settings.

## 2. Materials and Methods

### 2.1. Data Collection

We collected authoritative health information on anxiety disorders from the websites of federal and state health agencies and not-for-profit health promotion organisations in the U. S., Australia, Canada, and the United Kingdom. The total database contains 557 full-length articles including 270 (48.47%) original English materials associated with automatic translations to Spanish which contained misleading errors. We labelled these materials as positive or “risk” samples. Around 51.53% of the total samples we collected were original English texts whose automatic translation into Spanish using Google Translate did not contain any misleading information. The evaluation of the Spanish translations by Google was through the comparison of the original English texts with their backtranslations from Spanish. This method known as forward and backward translation was endorsed by international health organisations such as the World Health Organisation [15]. We subsequently labelled such English texts as negative or “safe” cases for automatic translation. We divided the entire database into 67.65% training data (389) and 32.35% testing data. Within the training dataset, there were 187 positive “risk” English texts which were prone to automatic translation errors and 202 negative “safe” English texts which had been proven to be suitable and reliable for automatic translation to Spanish. Similarly, within the testing dataset, there were 83 positive samples and 85 negative samples for testing the performance of classification of the classifiers to be developed. We applied 5-fold cross-validation on the training dataset to develop the classifiers to help remove biases in the development of algorithms.

### 2.2. Annotation of Feature Sets

Traditionally, health translation mistakes are believed to be associated with or triggered by the linguistic difficulty or lack of readability of the original English materials including complex, sophisticated, structural, syntactic, and lexical features. However, with the rapid developments of machine translation technologies, more research shows that semantic polysemy, that is, the multiple meanings of a certain word across domains and other issues, could be more challenging for latest neural machine translation tools. As a result, we included four large sets of features to investigate possible reasons which have triggered significant mistakes in machine-translated health materials from English to Spanish.

We annotated both training and testing datasets with 4 large sets of linguistic features: structural features (24 in total using Readability Studio software), lexical dispersion rates based on the British National Corpus (20 features in total), English lexical semantic features (115 features in total), which we annotated using the USAS system developed by the University of Lancaster [16–18], UK, and lexical sentiment features (92 features in total) that we annotated using Linguistic Inquiry and Word Count software. Appendix A shows the details of these 4 linguistic features.

### 2.3. Bayesian Machine Learning Classifiers (MLCs)

Bayesian MLC is a sparse classifier which can effectively counter model overfitting with relatively small datasets like ours. Bayesian classifiers are different from other supervised machine learning techniques in that they produce the posterior odds of a class dependent on the prior odds of an event and asymmetrical classification errors of the model, whereas frequentist ML classifiers only return a hard binary decision. In solving practical questions like ours, posterior odds are much more informative than a certain predicted binary outcome, as the Bayesian-style prediction using posterior odds helps practitioners and decision makers to appreciate the levels of risks of negative and positive cases over a continuous probability scale and assists in developing more effective intervention strategies to achieve optimal outcomes. This advantage of Bayesian MLs suits the purpose of our study, as we aimed to identify original English mental health materials which are more likely to cause significant errors if translated using automatic tools without further human evaluation. This can help health agencies developing bilingual health materials to better invest their resource and minimise risks of using machine translation in healthcare settings.

## 3. Methods

**Figure 1:**
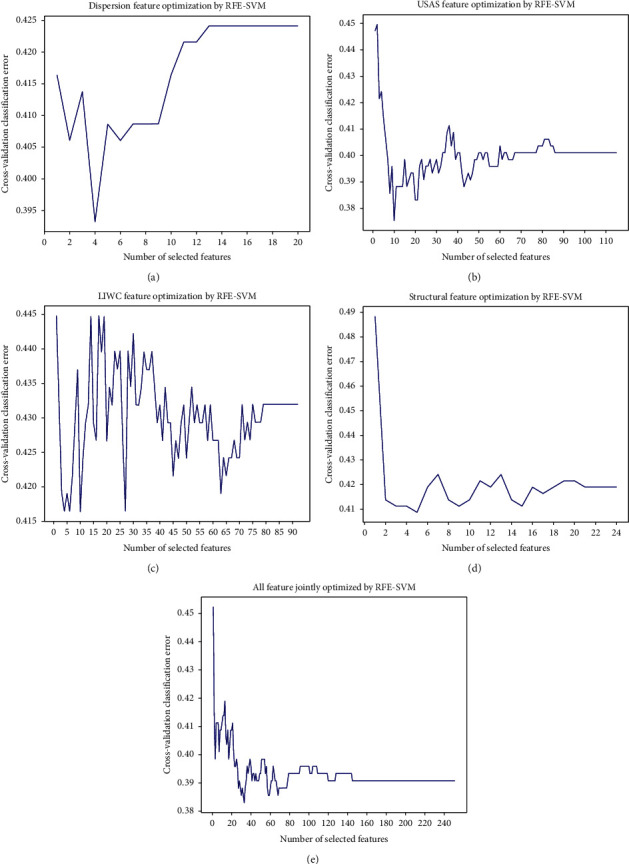
Automatic feature selection recursive feature elimination with SVM as base estimator.

To identify the best subset of features within each annotation category, as well as the best subset of features across annotation categories, we applied separate and combined feature optimisation techniques. The automatic feature selection technique we used was recursive feature elimination (RFE) with cross-validation in Python scikit-learn to increase the generalisability and accuracy of the Bayesian machine learning classifiers we developed. To identify and rank highly predictive features, recursive feature elimination used linear kernel support vector machine (SVM) as the base estimator. An optimal set of features was identified when the cross-validated classification error reached the minimal value.  Figure 1 shows the results of the automatic optimisation of different feature sets: in Figure 1(a), the optimised features of lexical dispersion rates were 4, as the cross-validated classification error dropped from 0.425 with the full feature set (20 in total) to 0.393 when the features were reduced to 4. In Figure 1(b), the optimised feature set of English semantic features was 10, as the cross-validated classification error decreased from 0.40 with the full feature set (115) to 0.375 when the features were reduced to 10. Further elimination of features however led to a spike in the classification error. In Figure 1(c), the optimised feature set of English sentiment features (annotated using the LIWC software) was 10, as we observed that the minimal classification error of 0.416 was reached when the total number of sentiment features was scaled back from 92 to 10. In Figure 1(d), 5 optimal structural features (92 in total) were identified when the minimal classification error of 0.409 was reached. Lastly, in Figure 1(e), we conducted the combined feature selection by integrating the 4 feature sets (251 in total): lexical dispersion rates and semantic, sentiment, and structural features. The optimal number of features emerged from the combined optimisation was 33 which was associated with the minimal classification error of 0.383.

## 4. Results and Discussion

### 4.1. Results

Following automatic feature optimisation to enhance the classification accuracy of classifiers, we evaluated the performance of Bayesian models (relevance vector machine (RVM)) with different feature sets on both the training and testing datasets. As discussed earlier, we applied 5-fold cross-validation on the training dataset to minimise biases in the classifiers being developed. First, we compared the original feature sets with their respective optimised feature sets in Table 1–5. Next, we compared the performance of RVM classifiers with different pairs of optimised feature sets.  Table 6 shows the comparison of RVM classifiers with double, triple, and quadruple optimised feature sets, respectively. Like feature optimisation, feature normalisation is another useful automatic technique to enhance the performance of machine learning classifiers. We applied three popular feature normalisation techniques: min-max, L_2_-norm (L2), and Z-score normalisation with each RVM classifier to see whether this could help balance asymmetrical classification errors within each model.

Table 1 shows the performance of RVM classifiers with lexical dispersion rates as features. It shows that after automatic feature selection, RVM with the reduced and optimised feature set (D4) reached a largely comparable performance to that of the classifier run on the full feature set: on the training dataset, the mean of area under the curve (AUC) of RVM (D4) was 0.617 (SD = 0.06), compared to 0.625 (SD = 0.06) of RVM (full 20 features), suggesting that feature reduction could also help encounter the issue of overfitting in training machine learning classifiers. On the testing dataset, the AUC of the RVM (D4) (0.648) was similar to that of RVM (All 20) (0.649). Sensitivity dropped slightly from 0.578 (RVM-All 20) to 0.566 (RVM-D4), and specificity remained unchanged at 0.753. Normalisation did not improve RVMs with the entire or optimised feature sets of lexical dispersion rates.

Table 2 compares the performance of RVM classifiers run on English semantic features. It shows that after automatic feature selection, the performance of the RVMs improved on both the training and the testing datasets: on the training data, the mean of AUC of RVM with the full semantic feature set (USAS115) observed a marginal improvement from 0.652 to 0.659 with a slightly reduced standard deviation (SD) from 0.052 to 0.045. On the testing dataset, the AUC of RVM (USAS115) saw an improvement from 0.692 to 0.714. Specificity improved from 0.729 of RVM (USAS115) to 0.777 of RVM (U10); sensitivity decreased from 0.590 of RVM (USAS115) to 0.578 of RVM (U10). Normalisation did not improve model performance.

Table 3 compares the performance of RVMs with English sentiment features annotated with the Linguistic Inquiry and Word Count (LIWC) software. It shows that after automatic feature optimisation, the performance of the RVM classifier (LWIC all 92) improved on the testing datasets. The AUC of RVM (L10) increased from 0.580 to 0.605. Model sensitivity increased from 0.518 to 0.651, but specificity decreased from 0.577 to 0.494. The impact of feature normalisation on RVMs with all and optimised feature sets was similar, while the classifier specificity improved, sensitivity decreased, and the overall model accuracy on the testing dataset however did not improve significantly.

Table 4 compares the performance of RVMs with various structural features which we annotated with the Readability Studio software. After automatic feature optimisation, the AUC of the classifier RVM (structural all 24) decreased from 0.636 to 0.621, which was due to decreased model sensitivity from 0.518 to 0.446, but the model specificity increased from 0.729 to 0.788. Feature normalisation helped to balance the asymmetric classification errors on the classifier RVM with both the entire feature set and the optimised feature set: the model specificity decreased and sensitivity increased. However, the overall model accuracy or the AUC did not improve with different feature normalisation techniques.

Table 5 shows the performance of the RVM with the combined feature sets of lexical dispersion rates and semantic, sentiment, and structural features, which represented 251 features in total. Automatic feature optimisation reduced the original feature set of 251 features to a parsimonious model containing 33 features only. With less predictive and noisy features involved in the model, the performance of the classifier also improved significantly on both the training and the testing datasets: on the training data, the model AUC was 0.642 (SD = 0.038). This increased to 0.658 (SD = 0.034) with the optimised RVM classifier. On the testing data, the AUC improved from 0.647 to 0.718. With automatic feature optimisation, both sensitivity and specificity improved: sensitivity increased from 0.518 to 0.554 and specificity increased from 0.706 to 0.741. Importantly, feature normalisation played a critical role in balancing asymmetrical classification errors on RVMs with combined feature sets. Specifically, min-max normalisation increased sensitivity of the optimised classifier to 0.651, the highest so far, and retained the high specificity of the classifier at 0.741. This sensitivity and specificity pair was the best combination among the classifiers developed so far.

**Figure 2:**
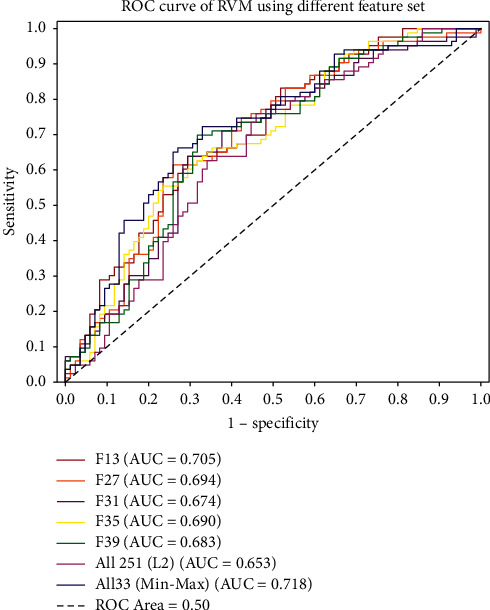
AUCs of RVMs on testing data using different feature sets.

Table 6 compares the performance of classifiers with double and multiple optimised feature sets. We compared in total 10 different pairs of optimised feature sets and conducted feature normalisation with each combination of optimised features, and as a result, each RVM in Table 6 has four different versions: the unnormalised version, followed by normalised versions with min-max, L2, and Z-score normalisation. The 10 combinations of optimised features were as follows: optimised lexical dispersion rates (D4) and optimised semantic feature (U10) (F1–F4), optimised lexical dispersion rates (D4) and optimised sentiment features (L10) (F5–F8), optimised lexical dispersion rates (D4) and optimised structural features (S5) (F9–F12), optimised semantic features (U10) and optimised sentiment feature (L10) (F13–F16), optimised semantic features (U10) and optimised structural features (S5) (F17–F20), optimised sentiment features (L10) and optimised structural features (S5) (F21–F24), and so on. We identified 5 high-performing models based on considerations of the overall model AUC, accuracy, sensitivity, and specificity: F13 was the unnormalised combination of optimised semantic (U10) and optimised sentiment features (L10). It had an overall AUC on the testing data of 0.705, with a relatively high sensitivity of 0.639 and specificity of 0.706. F27 was the normalised version (through L2 normalisation) of optimised lexical dispersion rates (D4), semantic features (U10), and sentiment features (L10). It had an overall AUC of 0.694 on the testing dataset, with sensitivity of 0.615 and specificity of 0.718. F31 was the normalised version (through L2 normalisation) of optimised lexical dispersion rates (D4), semantic features (U10), and optimised structural features (S5). It had an overall AUC of 0.674 on the testing dataset, with sensitivity of 0.639 and specificity of 0.671. F35 was the normalised version (through L2 normalisation) of optimised semantic features (U10), sentiment features (L10), and optimised structural features (S5). It had an overall AUC of 0.690 on the testing dataset, with sensitivity of 0.663 and specificity of 0.612. Finally, F39 was the normalised version (L2 normalisation) of optimised lexical dispersion rates (D4), semantic features (U10), sentiment features (L10), and optimised structural features (S5). It had an overall AUC of 0.683 on the testing dataset, with sensitivity of 0.627 and specificity of 0.694.  Figure 2 shows the visualised comparison of the AUCs of these 5 high-performing classifiers, the RVM with the entire combined feature sets (251 features) with L2 normalisation, and the best-performing classifier we developed (RVM (All33)) with min-max normalisation.

Tables 7 and 8 show the paired sample *t*-tests assessing the significance levels of differences in sensitivity and specificity between the various competing high-performance classifiers and the best-performing RVM classifier we developed through the combined automatic optimisation of four different feature sets followed by automatic feature normalisation. To reduce false discovery rates in multiple comparison, we applied the Benjamini–Hochberg correction procedure [19–21]. The results show that sensitivity of our best-performing RVM classifier was significantly higher than that of most other high-performing models, except for F35 (*p* = 0.0017); specificity of our best-performing RVM classifier was statistically higher than that of all other competing models with *p* values equal to or smaller than 0.004.

**Figure 3:**
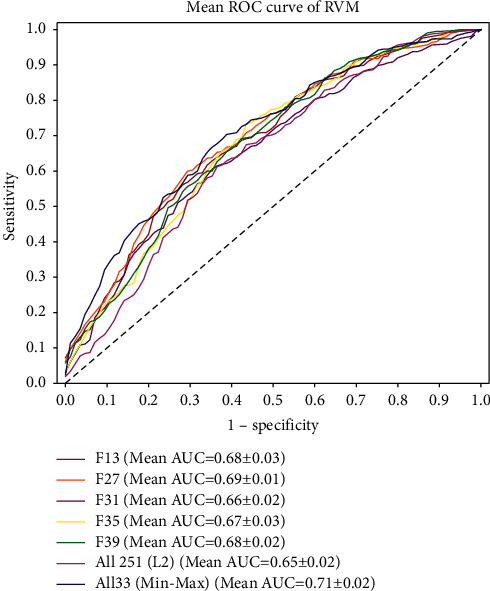
Mean AUCs of RVMs with different feature sets on testing data using different training dataset sizes.

Table 9 shows the paired sample *t*-tests assessing the significance levels of differences in AUCs between various competing high-performance classifiers and the best-performing RVM classifier on testing data using different training dataset sizes (i. e., 100, 150, 200, 250, 300, and all 389 training samples). We applied Benjamini–Hochberg correction to reduce false discovery rates in multiple comparison. The results show that AUC under different training dataset sizes of our best-performing RVM classifier was significantly higher than that of most other high-performing models, except for F13 (*p* = 0.0752) and F27 (*p* = 0.1698).  Figure 3 shows the visualised comparison of the mean AUCs of these 6 competitive classifiers and the developed best-performing classifier. As shown in Figure 3, our best-performing RVM classifier gained the highest mean AUC than all other competing models.

## 5. Discussion

A few important findings emerged in our extensive computational analyses, especially the search for the best subset of features for developing Bayesian machine learning classifiers to address our core research question, which was to predict and assess the risk levels of original English mental healthcare materials in terms of their suitability for automatic neural machine translation targeting Spanish-speaking patients. Our study shows that separate feature optimisation on the four distinct feature sets did not achieve acceptable pairs of model sensitivity and specificity. Let us take a close look at features retained in each optimised feature set.

Table 10 summarises the list of separately and jointly optimised features. First, the optimised feature set of lexical dispersion (D4) contained DiSp8: 0.7–0.8, DiSp9: 0.8–0.9, DiSp10:0.9–1.0, and DiWr10:0.9–1.0. Lexical dispersion rate is a measurement of familiarity of language to the public. We used existing lexical dispersion rates of the British National Corpus which had 10 intervals between 0 and 1 for spoken and written materials, respectively. In both spoken and written materials, higher lexical dispersion rates like those in the optimised feature set (D4) indicate that automatic machine translation mistakes were strongly associated with lexical items of higher familiarity in spoken and written materials. We used non-parametric independent sample test and Mann–Whitney *U* test to compare samples labelled as “risky” and “safe” original English mental health materials for automatic machine translation. The result shows that all 4 optimised lexical dispersion rates had statistically higher means in “risky” than in “safe” English mental health materials: DiSp8:0.7–0.8 (safe text class: mean (*M*) = 20.689, standard deviation (SD) = 11.963, standard error (SE) = 0.943; risky text class: *M* = 27.854, SD = 14.628, SE = 1.283, *p* < 0.0001), DiSp9:0.8–0.9 (safe texts: *M* = 44.553, SD = 15.679, SE = 1.236; risky texts: *M* = 54.400, SD = 17.275, SE = 1.515, *p* < 0.0001), DiSp10 : 0.9–1.0 (safe texts: *M* = 78.217, SD = 21.115, SE = 1.664; risky texts: *M* = 88.423, SD = 23.223, SE = 2.037, *p* < 0.0001), and DiWr10 : 0.9–1.0 (safe texts: *M* = 147.453, SD = 47.024, SE = 3.706; risky texts: *M* = 176.346, SD = 53.828, SE = 4.721, *p* < 0.0001).

With the optimised semantic feature set (U10), there were 10 semantic features identified as most relevant predictive features for the classifier. Similar to the optimised feature set of lexical dispersion rates (D4), the 10 optimised semantic features also had statistically higher means in potentially “risky” than in “safe” English mental health materials with regard to their suitability for automatic machine translation: A2 (changes, modifications) (safe texts: *M* = 15.429, SD = 15.093, SE = 1.190; risky texts: *M* = 24.185, SD = 20.646, SE = 1.811, *p* < 0.0001); A3 (existing status of objects, things, people) (safe texts: *M* = 37.267, SD = 28.570, SE = 2.252; risky texts: *M* = 57.062, SD = 41.987, SE = 3.682, *p* < 0.0001); A4 (classification) (safe texts: *M* = 4.311, SD = 5.299, SE = 0.418; risky texts: *M* = 8.062, SD = 10.504, SE = 0.921, *p* < 0.0001); A6 (comparison) (safe texts: *M* = 12.689, SD = 11.913, SE = 0.939; risky texts: *M* = 20.846, SD = 22.697, SE = 1.991, *p* < 0.0001); A7 (probability) (safe texts: *M* = 24.373, SD = 19.357, SE = 1.526; risky texts: *M* = 37.262, SD = 25.795, SE = 2.262, *p* < 0.0001); A13 (degree adverbs) (safe texts: *M* = 9.857, SD = 9.073, SE = 0.715; risky texts: *M* = 15.654, SD = 12.487, SE = 1.095, *p* < 0.0001); E5 (trepidation, courage, surprise) (safe texts: *M* = 3.901, SD = 6.927, SE = 0.546; risky texts: *M* = 11.515, SD = 18.045, SE = 1.583, *p* < 0.0001); O4 (physical attributes) (safe texts: *M* = 5.509, SD = 5.671, SE = 0.447; risky texts: *M* = 8.115, SD = 6.668, SE = 0.585, *p* < 0.0001); Z5 (functional words) (safe texts: *M* = 217.242, SD = 151.680, SE = 11.954; risky texts: *M* = 326.238, SD = 222.992, SE = 19.558, *p* < 0.0001); and Z6 (negative particles) (safe texts: *M* = 7.944, SD = 7.246, SE = 0.571; risky texts: *M* = 12.062, SD = 9.090, SE = 0.797, *p* < 0.0001).

Next, we examined the optimised feature set of English sentiment features (L10). This includes clout expressions (negative particles) (safe texts: *M* = 86.714, SD = 13.275, SE = 1.046; risky texts: *M* = 81.142, SD = 16.857, SE = 1.478, *p* = 0.004); emotional tones (safe texts: *M* = 29.106, SD = 32.179, SE = 2.536; risky texts: *M* = 18.367, SD = 27.907, SE = 2.448, *p* < 0.0001); words per sentences (safe texts: *M* = 18.714, SD = 5.075, SE = 0.400; risky texts: *M* = 19.728, SD = 4.813, SE = 0.422, *p* = 0.009); they (third person pronouns) (safe texts: *M* = 0.746, SD = 0.660, SE = 0.052; risky texts: *M* = 1.017, SD = 0.953, SE = 0.084, *p* = 0.028); affect words (safe texts: *M* = 8.581, SD = 2.517, SE = 0.198; risky texts: *M* = 9.382, SD = 2.604, SE = 0.228, *p* = 0.002); negative emotions (safe texts: *M* = 4.830, SD = 2.609, SE = 0.206; risky texts: *M* = 6.127, SD = 3.165, SE = 0.278, *p* < 0.0001); anxiety words (safe texts: *M* = 3.049, SD = 2.128, SE = 0.168; risky texts: *M* = 4.088, SD = 2.659, SE = 0.233, *p* < 0.0001); tentativeness words (safe texts: *M* = 5.043, SD = 1.643 SE = 0.129; risky texts: *M* = 5.599, SD = 1.590, SE = 0.139, *p* = 0.005); differentiation (safe texts: *M* = 4.176, SD = 1.515, SE = 0.119; risky texts: *M* = 4.717, SD = 1.333, SE = 0.117, *p* = 0.002); and core drives and needs (reward focus) (safe texts: *M* = 1.731, SD = 1.083, SE = 0.085; risky texts: *M* = 1.339, SD = 0.858, SE = 0.075, *p* = 0.001).

Within the optimised feature set of structural linguistic features (S5), there were 5 optimised features. Like the other three sets of optimised features, features retained in S5 had statistically higher means in “risky” texts than in “safe” English health texts: number of difficult sentences (more than 22 words) (safe texts: *M* = 10.491, SD = 8.821, SE = 0.695; risky texts: *M* = 16.200, SD = 14.048, SE = 1.23, *p* < 0.0001); number of monosyllabic words (safe texts: *M* = 560.186, SD = 358.796, SE = 28.277; risky texts: *M* = 811.446, SD = 515.400, SE = 45.204, *p* < 0.0001); number of long (6+ characters) words (safe texts: *M* = 280.255, SD = 215.525, SE = 16.986; risky texts: *M* = 439.215, SD = 347.782, SE = 30.502, *p* < 0.0001); number of sentences which use same words multiple times (safe texts: *M* = 10.814, SD = 12.263, SE = 0.966; risky texts: *M* = 19.177, SD = 22.999, SE = 2.017, *p* < 0.0001); and passive voice (safe texts: *M* = 2.944, SD = 5.639, SE = 0.444; risky texts: *M* = 4.931, SD = 6.191, SE = 0.543, *p* < 0.0001).

We found that the accuracy, sensitivity, and specificity of Bayesian classifiers based on these separately optimised features were suboptimal, in spite of the individual features retained in each optimised feature set being statistically significant features. Recent studies suggest that statistical significance and predictivity of features are often taken as exchangeable concepts, mistakenly [22–26]. Adding statistically significant features identified between case and control samples however do not necessarily improve the predictive performance of machine learning classifiers. This was verified in our study through joint optimisation of different feature sets combining lexical, semantic, sentiment, and structural features (251 features in total). The joint optimisation led to an optimised mixed feature set of 33 features, including 5 which did not have statistically different distribution in “safe” versus “risky” English mental health texts: K5 (leisure, activities) (safe texts: *M* = 2.596, SD = 5.473, SE = 0.431; risky texts: *M* = 2.292, SD = 2.900, SE = 0.254, *p* = 0.346); M3 (means of transport on land) (safe texts: *M* = 0.839, SD = 1.680, SE = 0.132; risky texts: *M* = 2.038, SD = 7.276, SE = 0.638, *p* = 0.076); Z3 (organisations names) (means of transport on land) (safe texts: *M* = 2.466, SD = 6.791, SE = 0.535; risky texts: *M* = 1.854, SD = 2.910, SE = 0.255, *p* = 0.893); average number of sentences per paragraph (safe texts: *M* = 2.160, SD = 3.394, SE = 0.267; risky texts: *M* = 2.434, SD = 6.268, SE = 0.550, *p* = 0.384); and number of questions (safe texts: *M* = 3.068, SD = 3.607, SE = 0.284; risky texts: *M* = 3.815, SD = 5.059, SE = 0.444, *p* = 0.312).

All other features in the jointly optimised feature set had statistically higher means in “risky” than in “safe” English mental health materials. Specifically, this included A3 (being/existing) (*p* < 0.001), A13 (degree adverbs) (*p* < 0.001), A15 (abstract terms of safety, danger) (*p* < 0.001), B2 (physical conditions) (*p* < 0.001), B3 (medical treatment) (*p* < 0.001), E5 (trepidation, courage, surprise) (*p* < 0.001), E6 (apprehension, confidence) (*p* = 0.001), M5 (transport by air) (*p* = 0.002), M6 (points of reference) (*p* < 0.001), N3 (measurement) (*p* = 0.004), O4 (physical attributes) (*p* < 0.001), S1 (social action, state, process) (*p* < 0.001), S5 (affiliation) (*p* = 0.001), T1 (time) (*p* < 0.001), X2 (reasoning, belief, scepticism) (*p* < 0.001), Z5 (functional words) (*p* < 0.001), Z8 (pronouns) (*p* < 0.001), clout expressions (*p* = 0.004), affect words (*p* = 0.002), negative emotions (*p* < 0.001), anxiety (*p* < 0.001), number of sentences using the same words multiple times (overused words) (*p* < 0.001), number of proper nouns (*p* = 0.008), number of unique multiple (3+) syllable words (*p* < 0.001), number of unique long (more than 6 letters) words (*p* < 0.001), and out-of-dictionary words (*p* = 0.004). For Bayesian machine learning classifiers to reach higher prediction accuracy, both these statistically significant features and statistically non-significant yet highly predictive features were identified “risk factors” contributing to the increased probability of conceptual mistakes in machine translated mental health information in Spanish.

The major advantage of the relevance vector machine classifier (RVM) based on the optimised and mixed feature set was the balanced sensitivity (0.651) and specificity (0.741), which made the instrument more applicable and useful in practical settings such as development and evaluation of mental health education and promotion resources for Spanish-speaking patients. The list of optimised linguistic features included in the best-performing classifier also provides important opportunities for health professionals to make well-targeted, cost-effective interventions to English health materials to improve their suitability for automatic translation purposes. For example, health professionals could adjust the distribution patterns of relevant linguistic features, especially those associated with higher risks of causing automatic translation mistakes, and rerun the automatic assessment of the English input materials using our machine learning classifier, iteratively, until the predicted risk level reaches an acceptable level. Importantly, this process does not require any linguistic knowledge on the part of English-speaking medical professionals of patients' language (in this case, Spanish).

## 6. Conclusions

Our paper developed probabilistic machine learning algorithms to assess and predict the levels of risks of using the Google Translate application in translating and delivering mental health information to Spanish-speaking populations. Our model can inform clinical decision making around the usability of the online translation tool when translating different original English texts on anxiety disorders into Spanish. This was achieved through the probabilistic prediction of Bayesian machine learning classifiers: if an input English text was assigned a high probability (over 50%) of causing erroneous and misleading automatic translation output, health professionals should become alert of the risk of using Google Translate; by contrast, if an input English text was assigned a low risk probability (below 50%), health professionals can feel reassured that the whole piece of English information can be translated safely to its intended user, using the online automatic translation tool. The smaller the risk probability of an English text is, the safer it is for the text to be translated automatically online. For original English materials which were labelled as non-suitable for automatic translation, our machine learning offers the opportunity to adjust, modify, and fine-tune the text to improve its suitability for automatic translation. This was achieved through the feature optimisation technique developed in our study. An important and useful feature of our model is that it does not require any linguistic knowledge on the part of English-speaking medical professionals of the patients' language. The classifier can be applied as a practical decision aid to help increase the efficiency and cost-effectiveness of multicultural health communication, translation, and education.

## Figures and Tables

**Table 1:** Performance of RVM classifiers with lexical dispersion features.

RVM	Training data		Testing data			
	AUC mean	SD	AUC	Accuracy	Sensitivity	Specificity
Dispersion rates (full 20 features)	0.625	0.06	0.649	0.667	0.578	0.753
Disp all 20 with min-max normalisation	0.547	0.04	0.654	0.643	0.566	0.718
Disp all 20 with L2 normalisation	0.573	0.07	0.594	0.560	0.518	0.600
Disp all 20 with Z-score normalisation	0.561	0.06	0.645	0.637	0.530	0.741
D4 (automatically optimised)	0.617	0.06	0.648	0.661	0.566	0.753
D4 with min-max	0.611	0.06	0.686	0.649	0.542	0.753
D4 with L2	0.571	0.07	0.595	0.560	0.518	0.600
D4 with Z-score	0.610	0.06	0.689	0.649	0.566	0.729

**Table 2:** Performance of RVM classifiers with lexical semantic features.

RVM	Training data		Testing data			
	AUC mean	SD	AUC	Accuracy	Sensitivity	Specificity
USAS all 115	0.652	0.052	0.692	0.661	0.590	0.729
USAS all 115 with min-max	0.593	0.082	0.677	0.643	0.578	0.706
USAS all 115 with L2	0.584	0.087	0.681	0.625	0.590	0.659
USAS all 115 with Z-score	0.589	0.111	0.694	0.655	0.639	0.671
U10 (automatically optimised)	0.659	0.045	0.714	0.679	0.578	0.777
U10 with min-max	0.663	0.044	0.723	0.679	0.578	0.777
U10 with L2	0.614	0.089	0.707	0.625	0.518	0.729
U10 with Z-score	0.646	0.042	0.713	0.649	0.506	0.788

**Table 3:** Performance of RVM classifiers with lexical sentiment features.

RVM	Training data		Testing data			
	AUC mean	SD	AUC	Accuracy	Sensitivity	Specificity
LIWC all 92	0.614	0.057	0.580	0.548	0.518	0.577
LIWC all 92 with min-max	0.577	0.054	0.646	0.619	0.566	0.671
LIWC all 92 with L2	0.610	0.064	0.573	0.548	0.518	0.577
LIWC all 92 with Z-score	0.619	0.046	0.670	0.619	0.506	0.729
L10 (automatically optimised)	0.602	0.040	0.605	0.571	0.651	0.494
L10 with min-max	0.629	0.055	0.607	0.566	0.566	0.565
L10 with L2	0.604	0.034	0.609	0.571	0.518	0.624
L10 with Z-score	0.614	0.068	0.616	0.583	0.578	0.588

**Table 4:** Performance of RVM classifiers with structural features.

RVM	Training data		Testing data			
	AUC mean	SD	AUC	Accuracy	Sensitivity	Specificity
Structural all 24	0.643	0.048	0.636	0.625	0.518	0.729
Structural all 24 with min-max	0.603	0.047	0.595	0.554	0.434	0.671
Structural all 24 with L2	0.647	0.046	0.616	0.595	0.590	0.600
Structural all 24 with Z-score	0.613	0.048	0.621	0.583	0.482	0.682
S4 (automatically optimised)	0.633	0.050	0.621	0.619	0.446	0.788
S4 with min-max	0.616	0.047	0.615	0.595	0.554	0.635
S4 with Z-score	0.624	0.044	0.603	0.601	0.542	0.659

**Table 5:** Performance of RVM classifiers with all (dispersion + USAS + LIWC + structural) features.

RVM	Training data		Testing data			
	AUC mean	SD	AUC	Accuracy	Sensitivity	Specificity
ALL 251	0.642	0.038	0.647	0.613	0.518	0.706
ALL 251 with min-max	0.626	0.041	0.697	0.643	0.590	0.694
ALL 251 with L2	0.670	0.045	0.653	0.619	0.639	0.600
ALL 251 with Z-score	0.633	0.085	0.680	0.625	0.590	0.659
ALL33 (automatically optimised)	0.658	0.014	0.670	0.649	0.554	0.741
ALL33 with min-max	0.678	0.034	0.718	0.696	0.651	0.741
ALL33 with L2	0.710	0.015	0.670	0.637	0.651	0.624
ALL33 with Z-score	0.672	0.036	0.682	0.643	0.627	0.659

**Table 6:** Performance of RVM classifiers with paired feature sets.

Feature	RVM	Training data		Testing data			
		AUC mean	SD	AUC	Accuracy	Sensitivity	Specificity
F1	*D*4 + U10	0.664	0.041	0.715	0.661	0.590	0.729
F2	D4 + U10 with min-max	0.639	0.041	0.736	0.661	0.566	0.753
F3	D4 + U10 with L2	0.673	0.034	0.681	0.643	0.578	0.706
F4	D4 + U10 with Z-score	0.627	0.065	0.722	0.649	0.554	0.741
F5	D4 + L10	0.650	0.041	0.681	0.595	0.615	0.577
F6	D4 + L10 with min-max	0.650	0.032	0.644	0.583	0.566	0.600
F7	D4 + L10 with L2	0.668	0.031	0.669	0.589	0.590	0.588
F8	D4 + L10 with Z-score	0.640	0.019	0.652	0.601	0.554	0.647
F9	D4 + S5	0.638	0.050	0.636	0.643	0.518	0.765
F10	D4 + S5 with min-max	0.648	0.041	0.650	0.625	0.566	0.682
F11	D4 + S5 with L2	0.655	0.054	0.608	0.607	0.602	0.612
F12	D4 + S5 with Z-score	0.580	0.035	0.584	0.566	0.482	0.647
F13	U10 + L10	0.670	0.058	0.705	0.673	0.639	0.706
F14	U10 + L10 with min-max	0.691	0.019	0.717	0.667	0.590	0.741
F15	U10 + L10 with L2	0.685	0.035	0.701	0.655	0.615	0.694
F16	U10 + L10 with Z-score	0.641	0.018	0.712	0.643	0.590	0.694
F17	U10 + S5	0.633	0.045	0.699	0.643	0.518	0.765
F18	U10 + S5 with min-max	0.627	0.031	0.713	0.661	0.542	0.777
F19	U10 + S5 with L2	0.588	0.078	0.617	0.566	0.458	0.671
F20	U10 + S5 with Z-score	0.633	0.030	0.704	0.643	0.566	0.718
F21	L10 + S5	0.671	0.055	0.668	0.631	0.554	0.706
F22	L10 + S5 with min-max	0.688	0.037	0.647	0.637	0.639	0.635
F23	L10 + S5 with L2	0.682	0.037	0.653	0.583	0.615	0.553
F24	L10 + S5 with Z-score	0.648	0.009	0.652	0.625	0.651	0.600
F25	D4 + U10 + L10	0.652	0.049	0.688	0.637	0.578	0.694
F26	D4 + U10 + L10 with min-max	0.674	0.041	0.658	0.601	0.518	0.682
F27	D4 + U10 + L10 with L2	0.689	0.047	0.694	0.667	0.615	0.718
F28	D4 + U10 + L10 with Z-score	0.669	0.033	0.653	0.613	0.554	0.671
F29	D4 + U10 + S5	0.649	0.041	0.697	0.649	0.566	0.729
F30	D4 + U10 + S5 with min-max	0.626	0.055	0.677	0.619	0.458	0.777
F31	D4 + U10 + S5 with L2	0.665	0.057	0.674	0.655	0.639	0.671
F32	D4 + U10 + S5 with Z-score	0.629	0.021	0.680	0.673	0.530	0.812
F33	U10 + L10 + S5	0.643	0.045	0.689	0.649	0.566	0.729
F34	U10 + L10 + S5 with min-max	0.685	0.027	0.697	0.637	0.590	0.682
F35	U10 + L10 + S5 with L2	0.663	0.054	0.690	0.637	0.663	0.612
F36	U10 + L10 + S5 with Z-score	0.667	0.018	0.696	0.637	0.627	0.647
F37	D4 + U10 + L10 + S5	0.622	0.033	0.657	0.643	0.578	0.706
F38	D4 + U10 + L10 + S5 with min-max	0.668	0.031	0.698	0.673	0.602	0.741
F39	D4 + U10 + L10 + S5 with L2	0.687	0.042	0.683	0.661	0.627	0.694
F40	D4 + U10 + L10 + S5 with Z-score	0.666	0.015	0.667	0.637	0.530	0.741

**Table 7:** Paired sample t-test of the difference in sensitivity between the best-performing model and other models.

No.	Pairs of RVMs	Mean difference	SD	95% CI		*p* value	Rank	(*i/m) Q*	Sig.
				Lower	Upper				
1	All33 (min-max) vs. F27	0.0361	0.0021	0.0319	0.0403	0.0012	1	0.0083	^*∗∗*^
2	All33 (min-max) vs. F39	0.0241	0.0015	0.0212	0.0270	0.0013	2	0.0167	^*∗∗*^
3	All33 (min-max) vs. All251 (L2)	0.0120	0.0008	0.0105	0.0136	0.0014	3	0.0250	^*∗∗*^
4	All33 (min-max) vs. F13	0.0120	0.0008	0.0105	0.0136	0.0014	4	0.0333	^*∗∗*^
5	All33 (min-max) vs. F31	0.0120	0.0008	0.0105	0.0136	0.0014	5	0.0417	^*∗∗*^
6	All33 (min-max) vs. F35	-0.0121	0.0009	−0.0137	−0.0104	0.0017	6	0.0500	^*∗∗*^

^∗∗^Statistical significance at 0.05 level using Benjamini–Hochberg correction procedure.

**Table 8:** Paired sample *t*-test of the difference in specificity between the best-performing model and other models.

No.	Pairs of RVMs	Mean difference	SD	95% CI		*p* value	Rank	(*i/m) Q*	Sig.
				Lower	Upper				
1	All33 (min-max) vs. All 251 (L2)	0.1412	0.0110	0.1195	0.1628	0.0020	1	0.0083	^*∗∗*^
2	All33 (min-max) vs. F35	0.1294	0.0105	0.1088	0.1500	0.0022	2	0.0167	^*∗∗*^
3	All33 (min-max) vs. F31	0.0706	0.0068	0.0573	0.0839	0.0031	3	0.0250	^*∗∗*^
4	All33 (min-max) vs. F39	0.0471	0.0048	0.0376	0.0566	0.0035	4	0.0333	^*∗∗*^
5	All33 (min-max) vs. F13	0.0353	0.0038	0.0279	0.0427	0.0038	5	0.0417	^*∗∗*^
6	All33 (min-max) vs. F27	0.0235	0.0026	0.0185	0.0286	0.0040	6	0.0500	^*∗∗*^

^∗∗^Statistical significance at 0.05 level using Benjamini–Hochberg correction procedure.

**Table 9:** Paired sample *t*-test of the difference in AUCs between the best-performing model and other models.

No.	Pairs of RVMs	Mean difference	SD	95% CI		*p* value	Rank	*(i/m) Q*	Sig.
				Lower	Upper				
1	All33 (min-max) vs F31	0.0482	0.0091	0.0304	0.0660	0.0000	1	0.0083	^*∗∗*^
2	All33 (min-max) vs. All 251 (L2)	0.0616	0.0279	0.0070	0.1163	0.0029	2	0.0167	^*∗∗*^
3	All33 (min-max) vs F39	0.0295	0.0165	−0.0028	0.0618	0.0071	3	0.0250	^*∗∗*^
4	All33 (min-max) vs F35	0.0304	0.0217	−0.0121	0.0728	0.0185	4	0.0333	^*∗∗*^
5	All33 (min-max) vs F13	0.0196	0.0214	−0.0224	0.0616	0.0752	5	0.0417	
6	All33 (min-max) vs F27	0.0138	0.0211	−0.0276	0.0552	0.1698	6	0.0500	

^∗∗^Statistical significance at 0.05 level using Benjamini–Hochberg correction procedure.

**Table 10:** Results of automatic feature optimisation.

Optimised features (number)	Label	Optimised feature
Lexical dispersion rates (4)	D4	DiSp8:0.7–0.8, DiSp9:0.8–0.9, DiSp10 : 0.9–1.0, DiWr10 : 0.9–1.0
Semantic features (10)	U10	A2 (words depicting change), A3 (words depicting being/existing), A4 (classification), A6 (comparing), A7 (probability), A13 (degree adverbs), E5 (trepidation, courage, surprise), O4 (physical attributes), Z5 (functional words), Z6 (negative particles).
Sentiment features (10)	L10	Clout expressions, emotional tones, words per sentences, they (third person pronouns), affect words (incl. positive and negative emotions, anxiety, anger, sad), negative emotions, anxiety, tentativeness, differentiation, core drives and needs (reward focus)
Structural features (5)	S5	Number of difficult sentences (more than 22 words), number of monosyllabic words, number of long (6+ characters) words, number of sentences which use the same word multiple times (overused words), passive voice
All features (33)	All33	Dispersion rates (1): DiSp8:0.7–0.8
		Semantic features (21): A3 (being/existing), A13 (degree adverbs), A15 (abstract terms of safety, danger), B2 (physical conditions), B3 (medical treatment), E5 (trepidation, courage, surprise), E6 (apprehension, confidence), K5 (leisure, activities), M1 (movement), M3 (transport on land), M5 (transport by air), M6 (points of reference), N3 (measurement), O4 (physical attributes), S1 (social action, state, process), S5 (affiliation), T1 (time), X2 (reasoning, belief, scepticism), Z3 (organisations names), Z5 (functional words), Z8 (pronouns)
		Sentiment features (L4): clout expressions, affect words, negative emotions, anxiety
		Structural features (S7): number of sentences using the same words multiple times (overused words), average number of sentences per paragraph, number of questions, number of proper nouns, number of unique multiple (3+) syllable words, number of unique long (more than 6 letters) words, out-of-dictionary words.

## Data Availability

The raw data supporting the conclusions of this article are available from the corresponding author upon request.

## Appendix

### A. Annotation of Feature Sets

#### A.1. Structural Features (24)

This includes average number of sentences per paragraph, number of difficult sentences (more than 22 words), average sentence length, number of interrogative sentence (question), number of exclamatory sentences, average number of characters, average number of syllables, number of numerals, number of proper nouns, number of monosyllabic words, number of unique monosyllabic words, number of complex (3+ syllable) words, number of unique 3+ syllable words, number of long (6+ characters) words, number of unique long words, out-of-dictionary words, repeated words, wording errors, redundant phrases, number of sentences which use the same word multiple times, wordy items, cliché, passive voice, and sentences that begin with conjunctions.

#### A.2. Lexical Dispersion Rates (20)

This includes DiSp1:0.0–0.1, DiSp2:0.1–0.2, DiSp3:0.2–0.3, DiSp4:0.3–0.4, DiSp5:0.4–0.5, DiSp6:0.5–0.6, DiSp7:0.6–0.7, DiSp8:0.7–0.8, DiSp9:0.8–0.9, DiSp10 : 0.9–1.0, DiWr1:0.0–0.1, DiWr2:0.1–0.2, DiWr3:0.2–0.3, DiWr4:0.3–0.4, DiWr5:0.4–0.5, DiWr6:0.5–0.6, DiWr7:0.6–0.7, DiWr8:0.7–0.9, DiWr9:0.8–0.9, and DiWr10 : 0.9–1.0.

#### A.3. English Lexical Semantic Features (115) (USAS)

This includes general and abstract terms (A1-A15, 15 features); the body and the individual (B1–B5, 5 features); arts and crafts (C1); emotion (E1-E6, 6 features); food and farming (F1–F4, 4 features); government and public (G1-G3, 3 features); architecture, housing and the home (H1–H5, 5 features); money and commerce in industry (I1–I4, 4 features); entertainment, sports and games (K1–K6, 6 features); life and living things (L1-L3, 3 features); movement, location, travel and transport (M1-M8, 8 features); numbers and measurements (N1–N6, 6 features); substances, materials, objects and equipment (O1–O4, 4 features); education (P1), language and communication (Q1-Q4, 4 features); social actions, states, processes (S1–S9, 9 features); time (T1-T4, 4 features); world and environment (W1–W5, 5 features); psychological actions, states and processes (X1-X9, 9 features); science and technology (Y1–Y2, 2 features); and grammar (Z0-Z9, Z99, 11 features).

#### A.4. Lexical Sentiment Features (92) (LWIC)

This includes analytical thinking (describing the degree to which people use words that suggest formal, logical, and hierarchical thinking patterns), clout (describing the relative social status, confidence, or leadership that people display through their writing or talking), authenticity (describing how people reveal themselves in an authentic or honest way), emotional tone (the higher the number, the more positive the tone; numbers below 50 suggest a more negative emotional tone), words per sentence), words longer than 6 letters, dictionary words, function words, pronouns, personal pronouns, personal pronouns (first per singular: I), personal pronouns (first person plural: we), personal pronouns (2nd person: you), personal pronouns (third person singular: she or he), personal pronouns (third person plural: they), impersonal pronouns, articles, prepositions, auxiliary verbs, common adverbs, conjunctions, negations, verbs, adjectives, comparatives, interrogatives, numbers, quantifiers, affect words, positive emotions, negative emotions, anxiety, anger, sad, social words, family, friends, female referents, male referents, cognitive processes, insight, cause, discrepancies, tentativeness, certainty, differentiation, perpetual processes, seeing, hearing, feeling, biological processes, body, health/illness, sexuality, ingesting, core drives and needs, core drives and needs (affiliation), core drives and needs (achievement), core drives and needs (power), core drives and needs (reward focus), core drives and needs (risk/prevention focus), past focus, present focus, future focus, relativity, relativity (motion), relativity (space), relativity (time), personal concerns (work), personal concerns (leisure), personal concerns (home), personal concerns (money), personal concerns (religion), personal concerns (death), informal speech, informal speech (swear words), informal speech (netspeak), informal speech (assent), informal speech (non-fluencies), informal speech (fillers), all punctuations, punctuations (periods), punctuations (commas), punctuations (colons), punctuations (semi colons), punctuations (question marks), punctuations (exclamations), punctuation (dash), punctuation (quotes), punctuation (apostrophes), parentheses (pairs), and other punctuations.
